# Triplex Real-Time RT-PCR for Severe Acute Respiratory Syndrome Coronavirus 2

**DOI:** 10.3201/eid2607.201285

**Published:** 2020-07

**Authors:** Jesse J. Waggoner, Victoria Stittleburg, Renee Pond, Youssef Saklawi, Malaya K. Sahoo, Ahmed Babiker, Laila Hussaini, Colleen S. Kraft, Benjamin A. Pinsky, Evan J. Anderson, Nadine Rouphael

**Affiliations:** Emory University, Atlanta, Georgia, USA (J.J. Waggoner, V. Stittleburg, R. Pond, Y. Saklawi, A. Babiker, L. Hussaini, C.S. Kraft, E.J. Anderson, N. Rouphael);; Stanford University, Stanford, California, USA (M.K. Sahoo, B.A. Pinsky)

**Keywords:** respiratory infections, severe acute respiratory syndrome coronavirus 2, SARS-CoV-2, SARS, COVID-19, 2019 novel coronavirus disease, coronavirus disease, zoonoses, viruses, coronavirus, rRT-PCR, molecular detection

## Abstract

Most reverse transcription PCR protocols for severe acute respiratory syndrome coronavirus 2 (SARS-CoV-2) include 2–3 targets for detection. We developed a triplex, real-time reverse transcription PCR for SARS-CoV-2 that maintained clinical performance compared with singleplex assays. This protocol could streamline detection and decrease reagent use during current high SARS-CoV-2 testing demands.

Detection of severe acute respiratory syndrome coronavirus 2 (SARS-CoV-2) typically relies on molecular testing of respiratory tract specimens, although viral RNA can be detected in other specimens (*1*). Real-time reverse transcription PCR (rRT-PCR) protocols have been described for SARS-CoV-2, but most involve testing with multiple, singleplex reactions (*2*–*6*). Such algorithms use large volumes of reagents and limit laboratory testing capacity, both of which have become crucial during the ongoing coronavirus disease pandemic (*7*). Multiplex assays are commercially available (*8*,*9*) but require specific platforms and are more expensive than laboratory-developed methods.

Our objective was to develop an internally controlled, triplex assay to detect SARS-CoV-2 RNA in clinical samples. We initially evaluated 6 individual rRT-PCRs, 3 published by the US Centers for Disease Control and Prevention (*2*) that target the nucleocapsid (N) gene, N1, N2, and N3; and 3 published by Corman, et al. (*4*) that target RNA-dependent RNA polymerase (RdRp), envelope (E), and N genes. We performed assays in 20 µL reactions of the Luna Universal Probe One-Step RT-qPCR Kit (New England Biolabs, https://www.neb.com) on a Rotor-Gene Q (QIAGEN, https://www.qiagen.com) by using 5 µL of eluate and our standard cycling protocol (*10*). We extracted total nucleic acids from samples on an EMAG (bioMérieux, https://www.biomerieux.com). We compared analytical sensitivity of the assays by using dilutions of 2 SARS-CoV-2 strains, BetaCoV/Germany/BavPat1/2020p.1 and USA-WA1/2020. The N2 and E-gene assays were the most sensitive singleplex reactions and we noted no substantial change in cycle threshold (C_t_) when the assays were combined. We then optimized a triplex assay to include the following targets: N2, which is SARS-CoV-2 specific; E, which also detects SARS-related coronaviruses; and RNase P, which serves as a heterologous, intrinsic specimen control (Appendix Table). We considered samples positive when they produced exponential amplification curves that crossed the threshold for both N2 and E targets.

The dynamic range of both SARS-CoV-2 targets in the triplex assay extended from 8.0 to 2.0 log_10_ copies/µL of eluate. We evaluated the lower limit of detection by performing serial dilutions of viral transport media (VTM) from a confirmed case by using VTM from confirmed negative cases. We tested eluates in quadruplicate and calculated RNA concentrations from a 4-point standard curve of quantified ssDNA (Integrated DNA Technologies, https://www.idtdna.com). The lowest concentration at which all replicates were detected by both targets was 45 copies/µL. When performed in singleplex, the N2 assay detected RNA down to 5 copies/µL, but all replicates had C_t_ >40, and the sensitivity of the E-gene assay did not change.

To evaluate specificity, we extracted total nucleic acids from 42 archived nasopharyngeal swab samples in VTM from patients who had laboratory-confirmed infections with the following viruses: other circulating coronaviruses in the United States (n = 20), influenza (n = 7), parainfluenza (n = 7), human rhinovirus (n = 6), respiratory syncytial virus (n = 3), human metapneumovirus (n = 3), and adenovirus (n = 2). Among the 42 swab samples, 6 had laboratory-confirmed co-infections with 2 viruses. All samples tested negative for both SARS-CoV-2 targets and positive for RNase P.

Finally, we tested nasopharyngeal or oropharyngeal swab samples from 27 patients with a suspected symptomatic SARS-CoV-2 infection (Table). Ten patients tested positive in the triplex assay. Results demonstrated 100% agreement with either the US Centers for Disease Control and Prevention or Corman et al. (*2*,*4*) protocols performed at CLIA-certified laboratories (Clinical Laboratory Improvement Amendments, https://www.cdc.gov/clia/about.html). Triplex results also agreed with testing in singleplex reactions except for 1 negative sample, number CoV 17, that gave a late positive signal in the N2 singleplex assay (C_t_ 44.8). However, no signal was detected in the E-gene singleplex. Therefore, had singleplex testing been performed, the final interpretation would not have differed.

**Table:** Comparison of cycle threshold results for 27 clinical samples tested in triplex real-time reverse transcription PCR and singleplex PCR for severe acute respiratory syndrome coronavirus 2

Sample	Triplex real-time reverse transcription PCR				Singleplex reactions	
	N2 target	E target	RNase P		N2 target	E target
No template control	ND	ND	ND		ND	ND
Positive control, RNA†	29.9	32.0	ND		30.7	33.0
CoV 01	19.0	23.1	21.0		19.2	23.1
CoV 02	ND	ND	22.1		ND	ND
CoV 03	ND	ND	22.0		ND	ND
CoV 04	ND	ND	21.9		ND	ND
CoV 05	ND	ND	22.4		ND	ND
CoV 06	ND	ND	21.4		ND	ND
CoV 07	ND	ND	21.6		ND	ND
CoV 08	26.1	28.0	25.5		26.1	27.7
CoV 09	20.1	22.0	22.5		20.3	22.2
CoV 10	ND	ND	21.1		ND	ND
CoV 11	ND	ND	22.0		ND	ND
CoV 12	ND	ND	20.5		ND	ND
CoV 13	ND	ND	20.9		ND	ND
CoV 14	ND	ND	24.6		ND	ND
CoV 15	ND	ND	28.7		ND	ND
CoV 16	14.6	16.3	22.0		14.9	16.5
CoV 17	ND	ND	27.6		44.8	ND
CoV 18	33.1	35.7	22.5		31.0	32.9
CoV 19	ND	ND	28.2		ND	ND
CoV 20	18.6	21.3	22.5		18.9	21.5
CoV 21	33.4	34.2	25.1		30.6	33.2
CoV 22	ND	ND	21.1		ND	ND
CoV 23	28.9	31.3	21.3		28.2	30.4
CoV 24	30.0	33.4	21.6		29.1	31.6
CoV 25	9.8	13.2	20.6		9.8	13.1
CoV 26	ND	ND	21.5		ND	ND
CoV 27	ND	ND	24.6		ND	ND

*CoV, coronavirus; E, envelope gene; N2, nucleocapsid 2 gene; ND, not detected; RNase P, ribonuclease P. †Strain BetaCoV/Germany/BavPat1/2020p.1.

We describe the development of an internally controlled triplex SARS-CoV-2 rRT-PCR that targets the N and E genes. The N2 and E-gene targets have proven to be sensitive in singleplex formats and assay performance remained robust to protocol changes we made during optimization in our laboratory. Of note, the triplex SARS-CoV-2 rRT-PCR has been validated only for the instruments and chemistries we describe here. This assay should be thoroughly validated before implementation in other laboratories.

Current molecular diagnostic workflows for SARS-CoV-2 contain 2 or 3 viral targets for confirmation (*2*–*6*). The triplex SARS-CoV-2 rRT-PCR we describe is consistent with this standard and demonstrated equivalent clinical performance to testing at CLIA-certified laboratories and to the component singleplex assays. In addition, the triplex format streamlines workflow and decreases reagent use. This triplex assay should, therefore, maintain accurate viral detection and improve laboratory capacity to meet the current high demand for testing.

Appendix Additional information on triplex real-time RT-PCR for SARS-CoV-2.
