# Supplementary material for: Triplex Real-Time RT-PCR for Severe Acute Respiratory Syndrome Coronavirus 2
Source: Emerg Infect Dis. 2020 Jul;26(7):1633–5. doi: 10.3201/eid2607.201285 (PMC7323516; doi:10.3201/eid2607.201285)
Supplement: Appendix — Additional information on triplex real-time RT-PCR for SARS-CoV-2. [file 20-1285-Techapp-s1.pdf]

Article DOI: https://doi.org/10.3201/eid2607.201285

Triplex Real-Time RT-PCR for Severe Acute Respiratory Syndrome Coronavirus 2

Appendix

Appendix Table. Primer and probe sequences and final reaction concentrations used to severe acute respiratory syndrome coronavirus 2 by triplex real-time reverse transcription PCR

Name	Sequence (5′→3′)	Concentration, nM*	Location†
2019-nCoV_N2-F	TTACAAACATTGGCCGCAAA	500	29164–29183
2019-nCoV_N2-R	GCGCGACATTCCGAAGAA	500	29213–29230
2019-nCoV_N2-P‡	ACAATTTGCCCCCAGCGCTTCAG	250	29188–29210
E_Sarbeco_F	ACAGGTACGTTAATAGTTAATAGCGT	400	26269–26294
E_Sarbeco_R	ATATTGCAGCAGTACGCACACA	400	26360–26381
E_Sarbeco_P1‡	ACACTAGCCATCCTTACTGCGCTTCG	200	26332–26357
RNase P – F	AGATTTGGACCTGCGAGCG	100	—
RNase P – R	GAGCGGCTGTCTCCACAAGT	100	—
RNase P – P‡	TTCTGACCTGAAGGCTCTGCGCG	50	—

*Concentration of each oligonucleotide in the final reaction mixture. RNaseP primer and probe concentrations were decreased to maintain consistent detection but minimize amplification in samples with high concentrations of human DNA.

†Genomic locations for viral primers and probes are provided based on the following reference sequences: Severe acute respiratory syndrome coronavirus 2 isolate 2019-nCoV/USA-IL1/2020, complete genome (Accession no. MN988713.1)

‡5′ fluorophore and 3′ quencher pairs were the following: 2019-nCoV_N2-P, FAM and BHQ-1; E_Sarbeco_P1, Quasar 670 and BHQ-2; RNase P – P, Cal Fluor Orange 560 and BHQ-1.
